# Dystonia and Cerebellum: From Bench to Bedside

**DOI:** 10.3390/life11080776

**Published:** 2021-07-31

**Authors:** Ryoma Morigaki, Ryosuke Miyamoto, Taku Matsuda, Kazuhisa Miyake, Nobuaki Yamamoto, Yasushi Takagi

**Affiliations:** 1 Department of Advanced Brain Research, Institute of Biomedical Sciences, Graduate School of Medicine, Tokushima University, Tokushima 770-8501, Japan; nyamamoto@tokushima-u.ac.jp (N.Y.); ytakagi@tokushima-u.ac.jp (Y.T.); 2 Department of Neurosurgery, Institute of Biomedical Sciences, Graduate School of Medicine, Tokushima University, Tokushima 770-8501, Japan; matsuda.taku@tokushima-u.ac.jp (T.M.); miyake.kazuhisa.2@tokushima-u.ac.jp (K.M.); 3 Department of Neurology, Institute of Biomedical Sciences, Graduate School of Medicine, Tokushima University, Tokushima 770-8501, Japan; ryom@tokushima-u.ac.jp

**Keywords:** dystonia, cerebellum, basal ganglia, pathogenesis, movement disorder, brain stimulation, plasticity, stress, animal models, phenomenology

## Abstract

Dystonia pathogenesis remains unclear; however, findings from basic and clinical research suggest the importance of the interaction between the basal ganglia and cerebellum. After the discovery of disynaptic pathways between the two, much attention has been paid to the cerebellum. Basic research using various dystonia rodent models and clinical studies in dystonia patients continues to provide new pieces of knowledge regarding the role of the cerebellum in dystonia genesis. Herein, we review basic and clinical articles related to dystonia focusing on the cerebellum, and clarify the current understanding of the role of the cerebellum in dystonia pathogenesis. Given the recent evidence providing new hypotheses regarding dystonia pathogenesis, we discuss how the current evidence answers the unsolved clinical questions.

## 1. Introduction

Dystonia is a clinical syndrome characterized by patterned, directional, sustained, or intermittent muscle contractions that cause abnormal dystonic postures and repetitive twisting dystonic movements [1,2,3,4]. The striking efficacy of pallidal deep brain stimulation suggests the involvement of the cortico-basal ganglia-thalamo-cortical feedback loop in its pathogenesis [5,6]. However, growing evidence extracted from basic and clinical research has additionally elucidated the importance of the cerebellum [5], suggesting that dystonia may arise from a motor network dysfunction, including both the basal ganglia and the cerebellum [7,8]. In this review, we collected research focusing on the relationship between “the cerebellum” and “dystonia”, trying to extract plausible answers to some of the unresolved clinical questions related to dystonia and aiming to gain a better understanding of its pathogenesis.

## 2. Neuroanatomical Consideration: Interaction between Basal Ganglia and the Cerebellum

Traditionally, the cortico-basal ganglia-thalamo-cortical and cortico-ponto-cerebello-thalamo-cortical loops are considered to be segregated, wherein the interaction between two loops occurs on thalamic relay neuron overlapping [9]. Currently, more direct communication between these two loops is considered to play a critical role in dystonia genesis [5].

The cerebellum can be grossly divided into three sagittal areas, which include the middle portion “vermin” or “vermal zone”, portions lateral to the vermis “the paravermis” or “the intermediate cortex”, and the most lateral parts “hemisphere” or “the lateral cortex” [10,11]. Purkinje cells, the only cerebellar cortex output elements, relay cerebellar cortex information on downstream deep cerebellar nuclei via GABAergic synaptic transmission. The cerebellum has four deep cerebellar nuclei, which lie on each side of the cerebellar midline. From medial to lateral, they are the fastigial, interposed (emboliform and globose), and dentate nuclei [12], which are targeted by the vermis, paravermis, and hemisphere, respectively [11,13]. These nuclei directly project to the thalamus, vestibular nuclei, inferior olive, red nucleus, locus coeruleus, anterior pretectal nucleus, and zona incerta [12,14]. Via the ventrolateral thalamus, these nuclei then connect to the frontal and parietal cortices, including the primary motor, prearcuate, premotor, and supplementary motor areas [13].

The cerebellum contributes to a feedforward system, which controls fast-coordinated movements [15]. Specifically, motor commands from the primary motor cortex to the spinal cord are copied and sent to the deep cerebellar nuclei, wherein the inferior olive nucleus receives predicted future outcome signals from the cerebellar nuclei [15]. The dentate nucleus receives input from the lateral cerebellar hemisphere; exerts a tonic facilitatory influence on downstream structures, controlling multi-joint fast movements [16], and is involved in planning, initiating, modifying voluntary movements, higher-level cognition, and sensory processing [17]. Meanwhile, the interposed and fastigial nuclei, situated in the spinocerebellum, are responsible for agonist–antagonist synergy (posture and gait), stretch reflexes, muscle tone, and slow single-joint movements [11,16]. The fastigial nuclei, especially the rostral division, are related to axial and proximal motor functions and encode the motion of the head and body in space [18,19,20,21].

The deep cerebellar nuclei have also been found to have a polysynaptic short-latency connection to the basal ganglia in rodents and non-human primates [22,23,24,25]. Dentate nucleus stimulation in cats evoked either bilateral caudate nucleus excitation or inhibition via the thalamic intralaminar nuclei [26]. Activation of the thalamic intralaminar centrolateral nucleus specifically induces metabolic contralateral deep cerebellar nuclei activation, and deactivation of the same nucleus elicits metabolic depression in the bilateral cerebellum (cortex and deep cerebellar nuclei) and basal ganglia [27] in rats, which implies functional connections. These nuclei also receive projections from the dorsal raphe nuclei serotonin neurons [28].

A study using retrograde tracing viruses in the monkey brain suggested that neurons in the dentate, interposed, and fastigial nuclei project mainly to D2-type medium spiny neurons in the putamen via the central lateral thalamic nuclei [22]. Other studies using antero- or retrograde tracing viruses in the rodent brain supported the notion that the cerebellar nuclei neurons project to D2-type medium spiny neurons in the dorsolateral striatum via the intralaminar or central lateral thalamic nuclei [23,29]. The intralaminar thalamic nuclei also have stronger innervation to striatal cholinergic and parvalbumin interneurons than cortices [30,31,32,33,34,35]. Furthermore, neurons in the non-human primate subthalamic nucleus (STN) have been found to have disynaptic innervation to the cerebellar cortex via the pontine nuclei or pedunculopontine nucleus, which is less prominent in rodents [24,36,37,38,39]. The red nucleus and zona incerta/field of Forel receive input from both the basal ganglia (entopeduncular nucleus, homologous to the primate globus pallidus interna) and cerebellum, which may integrate them [14,40,41]. In humans, a diffusion tractography study also delineated connections from the dentate nucleus to the basal ganglia, as well as from the subthalamic nucleus to the cerebellar cortex, as suggested by animal studies [42].

Somatotopic mapping is common throughout the cerebellum, including deep cerebellar nuclei, wherein the head is caudal, the tail rostral, the trunk lateral, and the extremities are medial [43]. The deep cerebellar nuclei projections innervate the premotor and motor cortices mainly through the ventrolateral thalamus [44,45,46,47], which comprises the ventral oral nucleus (Vo) and ventral intermediate nucleus (Vim). In particular, the ventrolateral thalamus has two subcortical afferent territories: the pallidothalamic and cerebellothalamic territories [48,49,50,51]. The pallidothalamic territory density decreases in an anterior (Vo side) to posterior (Vim side) gradient, whereas the cerebellothalamic territory density decreases in a posterior (Vim) to anterior (Vo) gradient [52]. Although the basal ganglia influence on supplementary motor areas is significantly greater than that of the cerebellum [53], cerebellar connectivity reduction induces a loss of inhibition in the sensorimotor and supplementary motor cortices [54]. Moreover, the cerebellum plays a role in proprioceptive information to M1 for sensing spatio-temporal aspects, which become deranged in dystonia [5,55]. Given that multiple structures, including the basal ganglia, cerebellum, thalamus, and sensorimotor cortex are disinhibited in dystonia [5], both the cortico-basal ganglia-thalamo-cortical and cortico-ponto-cerebello-thalamo-cortical loops seem to play critical roles in the pathogenesis of dystonia.

Neurons in mice deep cerebellar nuclei exert functional disynaptic innervation to the striatum via dopaminergic neurons in the ventral tegmental area [25]. The dopaminergic neurons in the ventral tegmental area innervate the nucleus accumbens and dorsal striatum [25,56,57]. Functionally, it is unclear whether neurons in deep cerebellar nuclei can directly modulate motor control via dopaminergic neurons in this area. Given that dopamine neurons in the substantia nigra compacta are primarily associated with motor function [58] and that the ventral tegmental area receives relatively little input from deep cerebellar nuclei [57,59], the cerebellum-ventral tegmental area-striatal pathway seems to have only a small contribution to basic motor control. In contrast, GABAergic neurons in the tail of the ventral tegmental area send inhibitory input to the dopaminergic neurons in the substantia nigra compacta, which may modulate motor function more efficiently [60]. Given that both dopaminergic and GABAergic neurons in the ventral tegmental area receive cerebellar afferents [25], cerebellar outputs may affect motor control via GABAergic neurons in the tail of the ventral tegmental area.

The brain loop consists of a continuous divergent-reconvergent architecture [61]. The anatomical hub structures should be a common reconvergent portion of the different loops. The primary hub structure of the two aforementioned loops is the thalamus. When we consider the third loop between the dopaminergic neurons in the substantia nigra compacta and striosome compartment in the striatum, the striatal interneurons could integrate the information from this loop and the thalamus.

## 3. Research Regarding the Role of the Cerebellum in Dystonia Genesis

### 3.1. Evidence from Animal Models of Dystonia

Morphological cerebellar abnormalities have been reported in rodent models of dystonia, such as *dt* rat, *tottering* mouse, leaner mouse, and Wriggle mouse Sagami [62,63,64,65,66]. Torsin A knockdown, for one, which targets the cerebellum but not the basal ganglia, induced dystonia in a mouse model of DYT1 [67]. Abnormalities in a restricted number of Purkinje cells were also found to be sufficient to cause generalized dystonia and more limited cerebellar regions of dysfunction-induced focal dystonia in mice [68]. Abnormal cerebellar activation was also evident in several different genetic rodent models of dystonia, including both transgenic and knockin DYT1 mice, dystonic (*dt*) rats, and *tottering* mice [8,68,69,70,71,72,73,74,75,76,77,78,79,80,81]. In addition, abnormal bursting of cerebellar Purkinje cells or neurons in the deep cerebellar nuclei was identified in rodent models of dystonia and pharmacological models of rapid-onset dystonia-parkinsonism [23,67,77,78,79,82], in which this abnormal cerebellar output drives abnormal high-frequency burst firing in the dorsolateral striatum [23]. Thus, eliminating cerebellar output reduces dystonic symptoms in these animals [8,23,28,74,75,83]. Additionally, abnormal cerebellar activation via the AMPA receptor agonists induces generalized dystonia in normal mice [84,85,86].

Notably, genetic silencing of the glutamatergic output of mice olivocerebellar fibers induces severe dystonia [87]. In contrast, 130-Hz inhibitory stimulation of bilateral interposed nuclei or centrolateral thalamic nuclei immediately abolished dystonia. Similarly, the dorsal raphe nuclei project 5HT-2A serotonergic fibers into the fastigial nucleus, and optogenetic photostimulation of this connection induces dystonia [28]. Thus, optogenetic photoinhibition of this connection or shRNA-mediated knockdown of the *ht2ar* gene in the fastigial nucleus was found to abolish dystonia in tottering mice [28]. These results might also explain the relationship between dystonia and stress.

Animal models of dystonia support the notion that cerebellar abnormalities, especially the hyperactivity of the cerebellar output, largely contribute to the genesis of dystonia. The clinical heterogeneity of dystonia suggests the involvement of multiple network malfunctions, and the cerebellum might be one of the key structures responsible for this heterogeneity.

### 3.2. Evidence from Clinical Research in Patients with Dystonia

In humans, dystonia has been reported in patients with cerebellar tumors, infarction, or spinocerebellar ataxia [88,89,90,91,92,93,94,95,96,97]. Dystonia patients with cerebellar atrophy have also been reported [98,99,100,101,102]. These overlapping phenomena of predominant dystonia and ataxia are called “predominant dystonia with marked cerebellar atrophy” or “slowly progressive cerebellar ataxia and cervical dystonia” [98,99,101]. Autopsy of cervical or generalized dystonia cases showed cerebellar abnormalities, including patchy loss [99,103], heterotopic existence, and dendritic swellings [104] of Purkinje cells. In contrast, a systemic review showed that up to 19% of patients with spinocerebellar ataxia (SCA) experienced dystonia during the overall disease course [105]. Dystonia is a relatively common manifestation of SCA2, 3, and 17 [106]. Approximately, 9–18.1% of SCA2, 24.2–24.6% of SCA3, and 52.7% of SCA17 patients manifest dystonia [105,107,108].

Cerebellar involvement in dystonia pathogenesis has been implicated in several imaging studies [62,109,110,111]. Morphologically, increased gray matter density has been observed bilaterally in the cerebellar flocculus or left cerebellum of idiopathic cervical dystonia patients [112,113] and in the bilateral cerebellum of patients with blepharospasm [113]. In contrast, a decrease in cerebellar gray matter in patients with writer’s cramp has also been reported [114]. An increase or decrease in the grey matter may indicate irritative or destructive lesions. These changes in the cerebellum have been considered secondary compensatory changes to the primary basal ganglia pathology; however, accumulating evidence suggests these cerebellar abnormalities are causal for dystonia genesis [115]. Abnormal cerebellar connectivity to the thalamus has been suggested in diffusion tensor imaging of DYT1 and DYT6 dystonia [116,117]. Several studies using positron emission tomography (PET) or functional magnetic resonance imaging have even shown increased cerebellar perfusion or metabolism [118,119,120,121,122,123,124,125,126,127,128]. A PET study using ^18^F-fluoroethoxybenzovesamicol, a radioligand of vesicular acetylcholine transporter (VAChT), showed that VAChT expression significantly decreased in the cerebellar vermis, which projects GABAergic output to the fastigial nucleus [129] in TOR1A/DYT1 patients as compared to the controls [130]. Moreover, pallidal deep brain stimulation reduces regional cerebral blood flow in the motor, premotor, prefrontal cortices, and cerebellum in tardive dystonia patients [128]. Collectively, it seems that hyperactivity in the cerebellar output might induce dystonia.

### 3.3. The Effect of Cerebellar Stimulation for Dystonia

Regarding non-invasive stimulation, transcranial magnetic stimulation [131,132,133,134] of the cerebellum temporarily alleviates dystonia, although the results of direct current stimulation [135,136,137] are controversial. Invasive cerebellar stimulation has been reported to be effective in secondary dystonia patients since the 1950s [138,139,140,141,142,143,144,145,146,147,148,149,150,151,152,153]. Cooper et al. used anterior lobe stimulation for cerebral palsy and dystonia [140,154,155]. Davis et al. and Galanda et al. have also used anterior cerebellar lobe or superior cerebellar peduncle high-frequency stimulations [142,143,144,146,147,148,149,150,151]. Although pallidal deep brain stimulation is still the gold standard for medically intractable generalized and cervical dystonia [156,157,158,159], Vo thalamic nucleus surgeries, which is innervated by both the pallidum and cerebellum, are effective for some forms of dystonia [160,161,162,163,164,165,166,167,168,169,170,171]. Recently, evidence from basic and clinical research has facilitated the revival of cerebellar surgery for dystonia [172,173,174,175,176,177,178], which mainly targets the deep cerebellar nuclei and superior cerebellar peduncles. The primary target nucleus in the cerebellum is the motor (dorsal) part of the dentate nucleus [173]. In addition, deep brain stimulation (DBS) of the superior cerebellar peduncle is preferred to avoid accompanying side effects, including dizziness, nystagmus, and ipsilateral leaning, as observed in studies of the dentate nucleus [172,175]. Recent studies have used high-frequency stimulations (104–300 Hz), pulse width (50–180 μs), 1.2–2.8V for stimulation of the dentate nucleus [174,178], and 130–200 Hz, 50–180 μs, 1.4–8.0 V for stimulation of the superior cerebellar peduncle [172,175,176]. Evidence also suggests the effectiveness of cerebellar modulation in dystonia treatment; however, the efficacy of GPi or Vo thalamic DBS, not the Vim nucleus, where more abundant cerebellar inputs come, implies the importance of the cortico-basal ganglia-thalamo-cortical circuitry in its pathogenesis [6]. Given that cerebral palsy patients respond well to cerebellar surgery, cerebellar DBS might be more effective for tonic-type dystonia and long-term illness-induced aberrant neuroplasticity as compared to pallidal DBS.

STN DBS is an interesting target for treating dystonia [179,180]. A recent meta-analysis comparing the efficacy of high-frequency STN DBS and GPi DBS suggested that STN DBS is more efficient at suppressing dystonia than GPi DBS in the long term [181]. STN DBS may modulate sensorimotor integration through orthodromic thalamocortical or antidromic hyperdirect pathway activation [182]. Delayed dystonia improvement after STN DBS may indicate the involvement of changes in disynaptic innervation from the STN to the cerebellar cortex via the pontine nuclei or pedunculopontine nucleus. In the future, combined pallidal, thalamic, subthalamic, and cerebellar DBS or personalized DBS treatment options may be considered in patients with various types of dystonia.

## 4. What Are the Roles of Two Distinct Loops?

The current concept of dystonia may answer some of its clinical questions. We can estimate the roles of two distinct loops, that is, the cortico-striato-pallido-thalamo-cortical and cortico-ponto-cerebello-thalamo-cortical loops. While the efficacy of pallidal-DBS in dystonia patients has been reported, it may take weeks to months to alleviate symptoms, and some patients, especially those who suffer from more chronic illnesses, manifest minimal improvement [111,183,184,185,186,187]. Delayed improvement has been established to be different from rapid improvement, as observed in Parkinson’s disease [115]. Another question is the type of dystonia. In human studies, dystonia patients usually show phasic or tonic dystonia, wherein pallidal-DBS has been found to be more effective for phasic dystonia and improves faster than tonic dystonia [156,186,188,189,190,191]. 

One hypothesis to explain this delay and insufficient efficacy after pallidal-DBS is that it takes time to modulate the rigid abnormal plastic change in the motor circuitry [111,115,163]. The neuronal activity of patients with dystonia is characterized by enhanced synchronized oscillations in the low-frequency band (4–12 Hz) [187,192,193,194,195,196,197,198]. It has been considered that the pathogeneses of phasic and tonic dystonia are different. Phasic dystonia is related to excessive resting-state pallidal low-frequency alpha oscillation and the cortico-striato-pallido-thalamo-cortical loop [187,194,199]. In contrast, tonic dystonia patients manifested resting-state pallidal delta oscillations, having no coherence with the motor cortex [187]. Liu et al. also showed pallidal low-frequency oscillatory local field potentials coherent with surface electromyograms (EMGs) in patients with phasic dystonia but not in those with tonic dystonia during involuntary dystonic movements [194]. These findings suggest that the mechanisms for developing phasic and tonic dystonic symptoms may differ at the basal ganglia level [194]. High-frequency pallidal-DBS suppresses pathological pallidal low-frequency activity and coherent EMG activity in patients with phasic dystonia [192,199]. Yokochi et al. further hypothesized that the cerebellothalamic pathway dysfunction induces tonic dystonia [187]. Frequent use of cerebellar surgery for dystonia in cerebral palsy patients suggests that tonic dystonia may arise from cerebellar dysfunction [173]. Additionally, DYT6 dystonia, which has been suggested to have abnormal cerebellar connectivity [116], is often treated unsatisfactorily with pallidal DBS [200,201,202]. Interestingly, a failed pallidal-DBS in a DYT6 dystonia patient was successfully treated with thalamic Vo-DBS in one study [163]. Clinically, we often observe both tonic and phasic components in patients with dystonia. Thus, the contribution of the cerebellum may differ from patient to patient depending on the underlying disorder.

Following DBS, tonic dystonia was found to improve slowly, possibly due to rigid maladaptive plasticity, which might partly be due to abnormal cerebellar activities from a loss of inhibition in the sensorimotor and supplementary motor cortices [5,54,109,203]. A high rate of recurrence in essential tremors after thalamic Vim-DBS [204,205,206] may partly support the hypothesis that cerebellothalamic connectivity dysfunction may induce rigid maladaptive plasticity [207].

Dopamine levels in the striatum should also be considered, given that either too little or too much of it can cause dystonia [208]. These two conflicting facts might be explained, in part, using the compartmental hypothesis [209,210,211]. Usually, hyperkinetic movement disorders, including phasic dystonia, are thought to be the consequence of excessive dopamine in the striatum. Given that Parkinson’s disease patients often experience off-tonic dystonia, and dopa-responsive dystonia (DYT5-GCH1) patients also manifest tonic dystonia, a lesser amount of dopamine is one of its causal factors [212]. Reduced dopamine level in the striatum with concomitant cerebellar abnormality induces dystonic movements in animal models [8] or human subjects [127]. Collectively, both loops might be involved in dystonia pathogenesis, wherein tonic dystonia might be more related to the cortico-ponto-cerebello-thalamo-cortical loop than the cortico-basal ganglia-thalamo-cortical loop.

## 5. Hypothesis for Dystonia Genesis

The reason why cerebellar lesions can cause ataxia and dystonia is still unclear. Prudente et al. hypothesized that destructive (suppressive) lesions are associated with ataxia, irritable (excitatory) lesions may cause dystonia, and these two could simultaneously exist in the cerebellum [115]. This hypothesis is consistent with prior studies suggesting that an increase in cerebellar output, such as an abnormal increase in Purkinje neuron firing or abnormal bursting patterns, can cause dystonia [109,115] and a strong relationship between dystonia and tremor [115]. Fremont and Khodakhah hypothesized that ataxia and dystonia exist on a continuum where modest changes in the regularity of cerebellar output underlie ataxia, while highly irregular firing (erratic bursting) cause dystonia [213]. Both theories could explain the coexistence of ataxia and dystonia due to cerebellar dysfunction.

**Figure 1:**
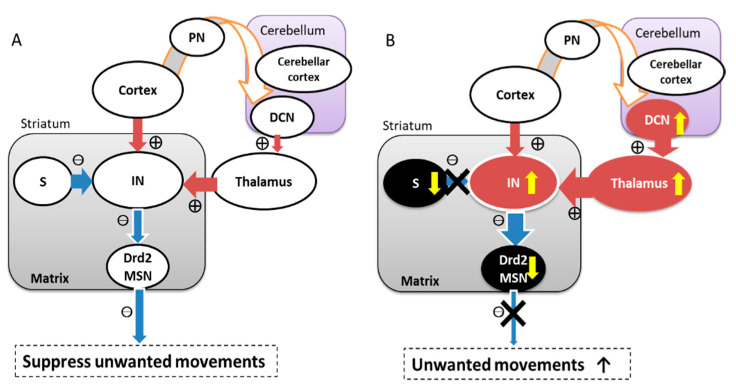
Possible hypothesis of the common mechanism underlying unwanted movements from several different sources. (**A**): normal condition, (**B**): aberrant condition; multiple structures (striosome, cortex, thalamus, cerebellum) can cause unwanted movements via hyperactivity of the cholinergic interneurons. S: striosome, IN: cholinergic interneurons, PN: pontine nucleus, DCN: deep cerebellar nuclei, Drd2 MSN: dopamine D2 receptor type medium spiny neurons.

We should separately evaluate two factors, that is, motor focusing and scaling [209,214]. The critical element for dystonia genesis might be the striatal switching system by the interneurons (Figure 1). In particular, cholinergic and parvalbumin neurons are important due to their powerful inhibition of medium spiny neurons [215,216]. When these interneurons depolarize, both D1 and D2 type medium spiny neurons are deactivated, resulting in direct pathway deactivation and indirect pathway activation. In contrast, interneuron hyperpolarization can activate both D1 and D2 type medium spiny neurons, thereby activating the direct pathways and deactivating the indirect pathways. Indirect pathway deactivation, that is, activation of D2 type medium spiny neurons, focuses the movements to be facilitated in an “intended” manner. Meanwhile, excessive indirect pathway deactivation, that is, excessive deactivation of D2 type medium spiny neurons, induces the loss of broad “unwanted movement” inhibition [217]. Through this switching system using the striatal interneurons, not only the striosome-matrix interconnection in the striatum [218], but also the cortico-striatal and thalamo-striatal innervation could change motor focusing.

Altered cholinergic transmission in a mouse model of dystonia has been reported [219,220]. Furthermore, the cholinergic interneurons are more influenced by the thalamo-striatal innervation than the cortico-striatal connection [30,31,32]. Repetitive stimulation from the intralaminar thalamus increases firing in the cholinergic interneurons [31], and clinically anticholinergic drugs are effective in dystonia patients [221]. The concept that cholinergic interneuron dysfunction induces motor focusing dysfunction in dystonia matches both hypotheses that dysfunction in the striosomes or the cerebellum causes dystonia (Figure 1) [222].

Similar to cholinergic interneurons, parvalbumin interneurons receive glutamatergic inputs from the cortex, centromedian, and parafascicular intralaminar nuclei [223,224,225], and thalamo-striatal synapses have a higher release probability on parvalbumin interneurons than cortico-striatal interneurons [35]. Animal studies showed that developmental delay in the maturation of parvalbumin interneurons causes dystonia in *dt^s^*^z^-mutant Syrian hamsters [226,227,228,229]. Injections of the parvalbumin interneuron inhibitor in the dorsolateral striatum elicit dystonia [230]. These thalamic or cortical modulations on the parvalbumin interneurons do not depend on dopamine or acetylcholine receptors [35]. Thus, the dysfunction of parvalbumin interneurons might determine the extent of abnormal movements.

**Figure 2:**
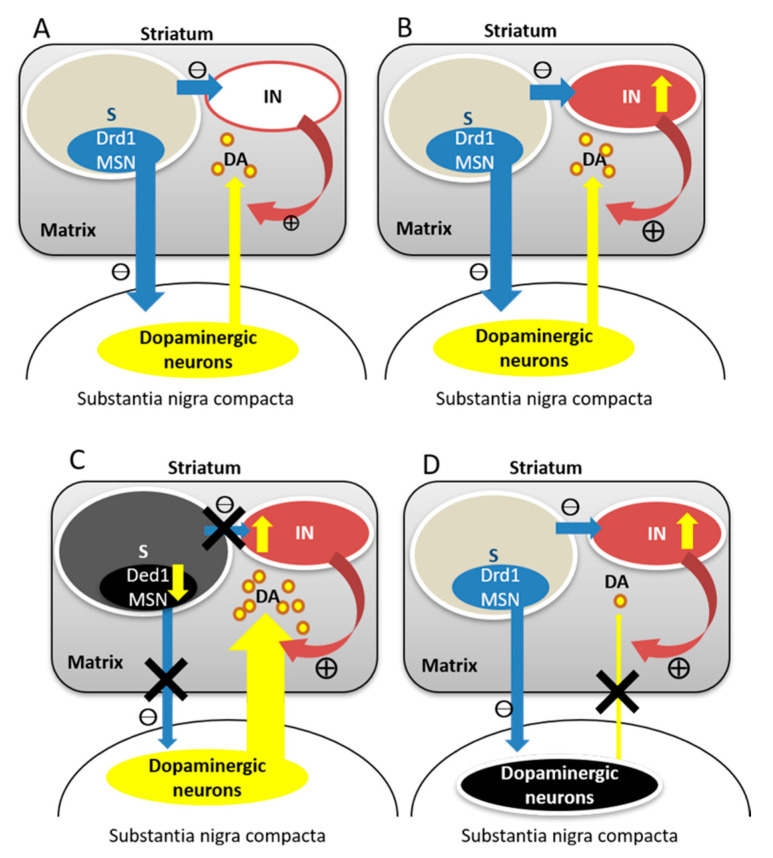
Hypothesis of excessive and insufficient dopamine releases in the striatum. (**A**): normal dopamine release, (**B**): increased dopamine release due to hyperactive cholinergic transmission, (**C**): excessive dopamine release due to striosomal dysfunction, (**D**): insufficient dopamine release due to dysfunction in the dopaminergic neurons. S: striosome, IN: interneurons, DA: dopamine, Drd1 MSN: dopamine D1 receptor type medium spiny neurons.

The amount of dopamine is probably related to movement scaling (Figure 2) [209]. Cholinergic interneuron activation can trigger dopamine release by activating presynaptic nicotinic receptors [231]. When striosome dysfunction occurs, dopamine may also be released via the striosomal circuit between the striosomes and substantia nigra compacta [210,211,232]. Striosomal dysfunction also activates cholinergic interneurons and facilitates dopamine release [231]. Excessive dopamine amounts lead to enlarging movements via direct pathway facilitation. In contrast, off dystonia in Parkinson’s disease and DYT5-GCH1 dystonia patients can be caused by the loss of suppression by the dopamine D2 receptor on the cholinergic interneurons due to decreased dopamine. In this context, a lesser amount of dopamine leads to minifying movements, that is, fixed or tonic dystonia, and thus the amount of dopamine in the striatum might determine the scaling of abnormal movements.

## 6. Concluding Remarks

Accumulating evidence suggests that the cortico-basal ganglia-thalamo-cortical, as well as cortico-ponto-cerebello-thalamo-cortical loops, are important in dystonia pathogenesis. The interaction between two loops, including each structure, may generate a differential dystonia phenotype. Despite these findings, its precise pathophysiology remains to be elucidated; however, recent evidence suggests that phasic dystonia may be more related to the basal ganglia circuitry, and tonic dystonia may be more affected by the cerebellar circuitry. Moreover, cerebellar circuitry abnormalities may induce rigid neuroplasticity. Specifically, striatal interneurons might be a key element in dystonia genesis, needing further studies to clarify the role of these loops in dystonia genesis. Accordingly, a new therapeutic option, that is, combined DBS or tailormade DBS, may be considered based on neuroimaging and neurophysiological findings in the future.

## Data Availability

Not applicable.
